# How Should Antibodies against *P. falciparum* Merozoite Antigens Be Measured?

**DOI:** 10.1155/2013/493834

**Published:** 2013-04-18

**Authors:** Sriwipa Chuangchaiya, Kristina E. M. Persson

**Affiliations:** Department of Microbiology, Tumor and Cell Biology (MTC), Karolinska Institutet, Nobels väg 16, 17177 Stockholm, Sweden

## Abstract

Immunity against malaria develops slowly and only after repeated exposure to the parasite. Many of those that die of the disease are children under five years of age. Antibodies are an important part of immunity, but which antibodies that are protective and how these should be measured are still unclear. We discuss the pros and cons of ELISA, invasion inhibition assays/ADCI, and measurement of affinity of antibodies and what can be done to improve these assays, thereby increasing the knowledge about the immune status of an individual, and to perform better evaluation of vaccine trials.

## 1. Introduction

Malaria kills around one million people every year [1, 2]. There is no vaccine against the disease, and resistance against medications is increasing. The symptoms of malaria include fever and anemia, and most of the deaths are caused by the parasite *Plasmodium falciparum*. The merozoite form of the parasite invades red cells, grows to form ring-, trophozoite- and schizont stages, and after rupture of the infected red cell new merozoites are released that are ready to enter uninfected red cells.

Merozoite invasion is a process that takes only a few minutes [3], but it involves several complex receptor-ligand interactions. Initial attachment of the merozoite is mediated by merozoite surface proteins such as MSP1 and MSP2, and is followed by reorientation of the merozoite where apical membrane antigen 1 (AMA1) is of importance [4, 5]. Other ligands such as erythrocyte-binding antigens (EBAs), for example, EBA140, EBA175, and EBA181 and *P. falciparum* reticulocyte-binding homologues (PfRhs), including PfRh1, PfRh2, PfRh4, and PfRh5 have also shown to be involved in the invasion process [6–9], even though the exact function of each antigen is not known. Genetic polymorphisms exist for many of the above-mentioned ligands, and based on some genes like MSP2, parasites can be grouped into two major allelic types: 3D7 and FC27. Serine repeat antigens (SERAs) are proteases that take part in forming a protein complex that is associated with the merozoite surface [10–12], and entry into the red blood cell is finally completed by an actin-myosin motor movement [13, 14].

Individuals who live in malaria endemic areas eventually develop immunity, but only slowly and after repeated exposure [15, 16]. Many of those that die of malaria are small children. During pregnancy, women have a greater risk of succumbing to malaria, and the fetus is also at risk [17]. Immunity against severe disease often develops before complete immunity is formed. It is known that antibodies are important in the defence against malaria, and it has been shown that passive transfer of antibodies from immune donors to individuals with *P. falciparum *infection reduces parasitemia and clears clinical symptoms [18–21]. However, exactly which specific antibodies are protective against future disease are not yet defined, and how they should be measured is even less clear. This information is urgently needed to be able to develop a functioning vaccine, something that has so far failed. We will here discuss the pros and cons for different methods available such as ELISA, invasion inhibition assays (IIAs), antibody-dependent cellular inhibition (ADCI), and affinity, and we compare it to measurements of antibodies in other diseases and how the overall evaluation of immunity or vaccine status of malaria could possibly be improved.

## 2. ELISA

When antibodies directed against different *P. falciparum* antigens have been measured, ELISA has usually been the method of choice. In this static method, proteins are coated to a plate and levels of antibodies in plasma from patients with (or without) malaria can be estimated. When recombinant proteins are used, only antibodies directed against specific antigens are analyzed. In real life, the antigen of choice is located together with several other antigens, for example, on the merozoite surface, and there might be interaction or competition between binding of different antibodies, something that is not accounted for in an ELISA. It has, for example, been shown for MSP1 that there are blocking antibodies that can compete with the binding of cleavage-inhibiting antibodies for epitopes on the merozoite [22, 23], and there are also other studies that have demonstrated that mixing of different antibodies can influence the outcome of the assay [24]. This kind of studies indicates that we should more look at using assays where the function of antibodies is studied, but the reason for why ELISAs are continued to be used to such a major extent is probably that they are very easy, fast, and robust to perform compared to functional assays.

When recombinant proteins are applied in ELISAs, the result might depend on which part of an antigen that is selected for use in the assay. For MSP1, it has been shown that antibodies against MSP1-19 were associated with some protection, while antibodies against MSP1 block-1 were not [25]. However, even when the same subdomain has been used such as in studies of EBA175, contradictory results have been achieved for whether there was a protective effect of antibodies or not [26, 27]. When red cells burst due to egress of merozoites, a lot of “debris” will be left in the blood stream that needs to be removed, and many of the antibodies might help in doing this but this does not mean that the antibodies will protect from future disease. If only ELISAs are used, it is difficult to discern which antibodies are functionally important.

In general, higher levels of antibodies are found in ELISAs in older individuals in endemic areas, while lower levels of antibodies are seen in younger individuals in the same areas. This was recently shown for the EBAs for example [28] and it has been shown earlier for other antigens as well [7, 26, 27, 29, 30]. However, even though an individual has high levels of antibodies, they can still develop malaria, and an individual with relatively low levels of antibodies can be fully protected from clinical and severe malaria [25, 31–52]. In vaccine trials, antibodies measured by ELISA have been shown to often be short-lived, and most patients will still get malaria in spite of presence of antigen-specific antibodies [53]. From a population perspective, ELISAs can be used to make an overall estimation of how much exposure there has been to malaria, but for each individual it is not possible to make an exact determination of the immune status. The only thing that can for sure be concluded from a positive response in ELISA is that the individual has at some stage during his/her lifetime been exposed to malaria.

When ELISAs against different antigens are combined, more information can possibly be acquired about the level of immunity in investigations of the breadth of antibodies [27], but the question of whether it is just a measure of exposure will still remain. A way of improving the ELISAs would be to more often use standardized controls, allowing for the measurement of exact amounts of specific antibodies instead of titers.

In conclusion, ELISAs are easy and robust to perform, and they can clearly give us information about whether or not an individual has ever been exposed to malaria. With combinations of different antigens and standardization of the assays, more information can possibly be provided. However, ELISAs do not tell us anything about the function of the antibodies, and on an individual level, ELISAs will not give us complete information about immunity.

## 3. Growth Inhibition Assay/Invasion Inhibition Assay

One assay that has been used to try and better determine the function of antibodies in plasma is growth inhibition assay or invasion inhibition assay (IIA). Antibodies directed against merozoite antigens are thought to function by directly inhibiting invasion of new red cells, which will then stop further multiplication of parasites, or through ADCI. By adding immune plasma, which contains antibodies to growing parasites, the inhibitory function of the antibodies can be evaluated in comparison to parasites where no plasma has been added. The downside of IIAs compared to ELISAs is that they are much more labor intensive, but on the other hand all proteins are expressed in their native environment and many both known and unknown potential interacting factors are included in the assay. There have been several studies that have shown invasion inhibitory activity of antibodies from human plasma, both when total IgG has been used and when malaria-antigen-specific fractions have been used [22, 54–58]. Some studies have shown increasing invasion inhibition with age [59] while others have shown more invasion inhibition in children [56, 60]. This kind of contradictory results might be explained by different functions in the antibodies repertoire being important during development of immunity, compared to when immunity is already established, but it might also mean that the assay is not yet fully optimized to show who is immune or not. One attempt to improve the IIA is by adding monocytes (ADCI). When ADCI has been employed, some antigens like MSP3 and GLURP [61, 62] have shown an inhibitory effect only when monocytes were included in the IIA. However, with ADCI there seems to be a major variability in the assay that can be seen from day to day even using the same donor of monocytes, making it difficult to standardize the assay [63]. If this assay could be improved and standardized, it might add very valuable information about different antibodies.

Another attempt to try and improve IIA is to use knockout lines of parasites. Here, a single antigen can be studied in its natural environment and with the correctly folded protein, and a comparison can be made between the wild-type and the knockout parasites. This has been used, for example, for the EBAs, where it was shown that antibodies against EBA175 was responsible for a major part of the inhibitory activity in some individual plasma samples, while other samples seemed not to have any functional antibodies against this protein at all [29]. This kind of results is important for selection of potential vaccine candidates, especially for ruling out those antibodies that have no effect. Some antigens have been difficult to knock out, in which case other reagents might have to be added to the assay such as blocking agents, to find out which antibodies are causing the inhibitory effect.

In conclusion, IIAs are labor intensive but can provide important information especially for comparisons between knockout and wild-type parasites, where the function of a single potential vaccine candidate antigen can be evaluated.

## 4. Affinity

Another way of looking at antibodies against merozoite antigens is to study the affinity/avidity of antibodies. Some studies have tried to use ELISA with added NH_4_SCN to evaluate affinity, but the results for this have been inconsistent [64, 65]. A new way of investigating affinity for vaccine trials has opened up with methods like surface plasmon resonance (SPR) [66, 67], where affinity of antibodies can be estimated under flow, something that ought to be more similar to the physiological situation compared to static assays. With this method, association and dissociation of antibodies binding to their target antigens can be studied in real time. This method has before mainly been used with monoclonal antibodies in malaria research, but it has recently been applied also to polyclonal antibodies. It has, for example, been shown that affinity of antibodies against AMA1 increased with age, and the presence of high affinity antibodies in plasma against MSP2-3D7 was associated with protection against malaria [68]. In this study, most plasma samples showed a relatively rapid on-rate, indicating that whether the concentration of antibodies in the samples is high or low, the antibodies will still bind quite fast to their antigens. This is important for considering whether an antibody will function in inhibiting merozoite invasion or not, since invasion is a process that takes only a couple of minutes. The dissociation rate (which is concentration independent) might therefore be more important for whether an antibody will function or not. In the referred study, monoclonal antibodies were also used and it could be seen that some bound with so low dissociation rates, that a value for the dissociation rate could not be obtained. These antibodies will probably have difficulties in inhibiting invasion. However, investigations of affinity of antibodies in malaria are yet a very new field which needs a lot more studies to be able to make firm conclusions, and standardized protocols needs to be in place to facilitate interpretation of the results. In other infectious diseases, such as bacterial diseases [69, 70], toxoplasma [71], or HIV [72], a lot more has been done in the field of affinity.

In conclusion, studies of affinity of polyclonal antibodies in malaria is a new field that could add a lot of information both about how immunity is formed and for vaccine trials, and more work in this area is needed.

## 5. Comparing Different Ways of Measuring Antibodies

**Figure 1:**
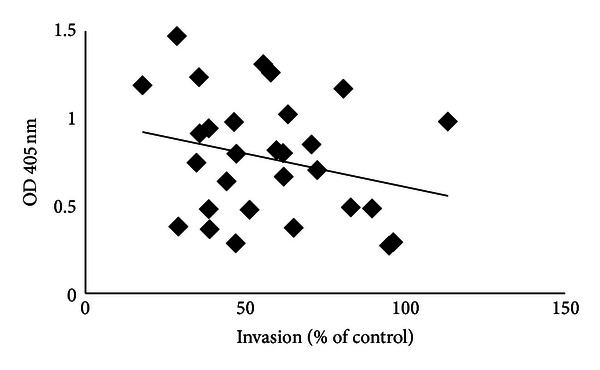
In a study of children with mild malaria in Uganda, IIA of a wild isolate was compared to ELISA using MSP2-3D7 as antigen *R* ^2^ = 0.07.

When different assays are used to evaluate antibodies against malaria, the methods often show results that do not correlate with each other. For example, SPR has been shown to correlate with ELISA for AMA1, which binds with relatively high affinity, but not for MSP2, which binds with lower affinity [68]. When IIA has been compared to ELISA, some people have shown correlations while others have not seen any correlations [36, 73]. This is probably because IIA is a functional assay, while ELISA only estimates the levels of antibodies. An example of the lack of major correlations between methods (IIA and ELISA) is shown in Figure 1. This illustrates the difficulties in estimating immunity against malaria. For vaccine trials, one has to be careful with interpretation of results, as the results could vary a lot depending on which method is used. In other fields such as HIV, international consortia have established common standards to be used in immunological assays, and this might be applicable for ELISAs, but more challenging for the functional methods yet available in malaria.

## 6. Which Method Should We Use to Measure Antibodies against Malaria?

None of the methods described here are good enough on their own to give a complete picture of an individual's immune status. ELISAs are good at giving us information about whether any immune response at all is mounted against a potential vaccine candidate, and from a population perspective when many samples need to be analyzed, ELISAs are easy to perform. However, if we want to know something about the function of a specific antibody, IIA with the usage of knockout and wild-type parasites should be the way forward. Even though these assays are more cumbersome to perform, they will add valuable information. Affinity of antibodies has so far been very scarcely studied in malaria research compared to many other diseases, and expansion of this field should add important information both for knowledge about immunity and for vaccine trials. More studies are needed that employ different methods together in the same patient cohorts to get a more full picture of which functions of antibodies are important during different stages of development of immunity against malaria.

In conclusion, which method should be used depends on what we want to know about the antibodies. If we only want to know whether antibodies are formed or not, ELISAs can be used, but if we want to know something about the function of the antibodies, more elaborate assays such as IIA have to be applied. To get the full picture about immunity status in an individual, the methods available have to be developed more and probably combined to a bigger extent, but with new methods such as those available for affinity measurements there is hope that this situation can be improved.
